# Ileal Perforation Caused by the Accidental Ingestion of a Press-Through Package in an Elderly Patient With Dementia: A Case Report

**DOI:** 10.7759/cureus.114270

**Published:** 2026-08-10

**Authors:** Takahiro Inoue, Hiroaki Kobayashi

**Affiliations:** 1 Gastrointestinal Surgery, Sapporo Kojinkai Memorial Hospital, Sapporo, JPN

**Keywords:** accidental ingestion, blister pack, computed tomography, dementia, elderly, ileal perforation, press-through package, ptp

## Abstract

A woman in her 90s who lived alone and had a history of unspecified dementia and pulmonary tuberculosis presented with a four-day history of lower abdominal pain. Her only regular medication was diazepam, which she dispensed from a press-through package (PTP) and had been cutting individual tablets from the sheet using scissors. Upon admission, she was hypothermic (body temperature 34°C), with otherwise preserved vital signs (blood pressure 131/65 mmHg, heart rate 79 beats/min, SpO₂ 98% on room air). She had no recollection of having swallowed a PTP. Laboratory findings showed a markedly elevated C-reactive protein level of 32.58 mg/dL, severe hypoalbuminemia (albumin 2.4 g/dL), and mild metabolic acidosis (pH 7.300, HCO₃⁻ 17.6 mmol/L) on arterial blood gas analysis. Abdominal computed tomography (CT) revealed a high-density object surrounded by a low-density halo in the ileum approximately 10 cm proximal to the ileocecal valve, along with localized free air and abscess formation in the right lower abdomen, consistent with a PTP-induced ileal perforation. Emergency laparotomy revealed a spindle-shaped ileal perforation, with an intact PTP sheet partially extruding through the defect. An abscess had formed between the abdominal wall and the bowel at the perforation site. Primary repair, omental reinforcement, and peritoneal lavage with drainage were performed without bowel resection or stoma creation. The patient recovered without complications and was discharged on postoperative day 15. This case illustrates two key points: the characteristic CT appearance of a retained PTP can confirm the diagnosis even without an ingestion history, and occult PTP ingestion should be considered in elderly patients with dementia who present with acute abdomen, irrespective of whether an ingestion history can be elicited.

## Introduction

Press-through packages (PTPs), or blister packs, are the primary method of oral tablet packaging in Japan and several other Asian countries. A PTP features a plastic dome containing a single tablet sealed against an aluminum foil backing. To minimize the risk of accidental ingestion of a small PTP unit, Japanese manufacturing standards mandate that perforation lines span at least two tablets, making it impossible to separate a single tablet by hand. However, patients who prefer to take tablets individually, either to avoid missed doses or for easier daily management, often cut tablets from the sheet with scissors, creating fragments with sharp edges that can penetrate the intestinal wall [1,2].

Ingested foreign bodies typically cause perforations at narrow or angulated anatomical sites in the gastrointestinal tract, such as the cricopharyngeus, the lower esophagus, and the ileocecal junction [3]. Diagnosing PTP ingestion is particularly challenging because the event is often unwitnessed, and patients, especially those with cognitive impairment, may be unable to provide a reliable history, and free intraperitoneal air is not always present [1,2]. Abdominal computed tomography (CT) can identify a retained PTP by revealing its characteristic appearance, a round, high-density tablet surrounded by a low-density halo from the air pocket within the dome; however, this finding is often overlooked without a history of ingestion [1,4,5].

We report a case of ileal perforation due to occult PTP ingestion in an elderly woman with dementia who was living alone, in whom CT confirmed the diagnosis despite her unawareness of having swallowed a PTP.

## Case presentation

A woman in her 90s with unspecified dementia and a past history of pulmonary tuberculosis presented to the emergency department with a four-day history of lower abdominal pain. She lived alone and was functionally independent. Her only regular medication was diazepam, dispensed in PTP format. She did not recall having swallowed a PTP and denied foreign body ingestion. Physical examination revealed hypothermia (body temperature 34°C), a blood pressure of 131/65 mmHg, a heart rate of 79 beats/min, an SpO₂ of 98% on room air, and intact consciousness (Glasgow Coma Scale score of 15). Abdominal examination revealed right lower quadrant tenderness and signs of peritoneal irritation without muscular guarding.

Laboratory findings revealed an elevated C-reactive protein of 32.58 mg/dL and a low albumin of 2.4 g/dL. Arterial blood gas analysis revealed mild metabolic acidosis (pH 7.300, HCO₃⁻ 17.6 mmol/L, base excess -8.9 mmol/L) with a lactate of 13 mg/dL, indicating preserved end-organ perfusion. Elevated fibrinogen levels (650 mg/dL) and fibrin degradation products (22.5 μg/mL) were consistent with a systemic inflammatory response, with no clinical or laboratory signs of overt disseminated intravascular coagulation. Complete laboratory data are provided in Table 1.

**Table 1:** Laboratory findings on admission Abnormal values are indicated by ↑ (above the reference range) or ↓ (below the reference range). BE: base excess; WBC: white blood cell count; Hb: hemoglobin; Hct: hematocrit; BUN: blood urea nitrogen; AST: aspartate aminotransferase; ALT: alanine aminotransferase; T-Bil: total bilirubin; CRP: C-reactive protein; PT-INR: prothrombin time-international normalized ratio; APTT: activated partial thromboplastin time; FDP: fibrin degradation products

Laboratory test	Value	Reference range
Arterial blood gas (room air)		
pH	7.300 ↓	7.350-7.450
pCO₂ (mmHg)	36.5	32.0-45.0
pO₂ (mmHg)	105.5	83.0-108.0
HCO₃⁻ (mmol/L)	17.6 ↓	21.0-28.0
BE (mmol/L)	-8.9 ↓	-2.0 to +2.0
Lactate (mg/dL)	13	4-14
Complete blood count		
WBC (×10³/μL)	9.1 ↑	4.0-8.0
Hb (g/dL)	11.7 ↓	12.0-16.0
Hct (%)	36.2	36.0-48.0
Platelets (×10⁴/μL)	23.8	12.0-40.0
Chemistry		
Total protein (g/dL)	5.4 ↓	6.7-8.3
Albumin (g/dL)	2.4 ↓	3.8-5.3
BUN (mg/dL)	47.6 ↑	8.0-20.0
Creatinine (mg/dL)	0.76	0.50-0.86
AST (U/L)	71 ↑	8-38
ALT (U/L)	36	4-44
T-Bil (mg/dL)	0.7	0.2-1.2
CRP (mg/dL)	32.58 ↑	≤0.30
Coagulation		
PT-INR	1.00	0.85-1.15
APTT (sec)	25.7	23.0-35.0
Fibrinogen (mg/dL)	650 ↑	200-400
FDP (μg/mL)	22.5 ↑	<5.0

**Figure 1:**
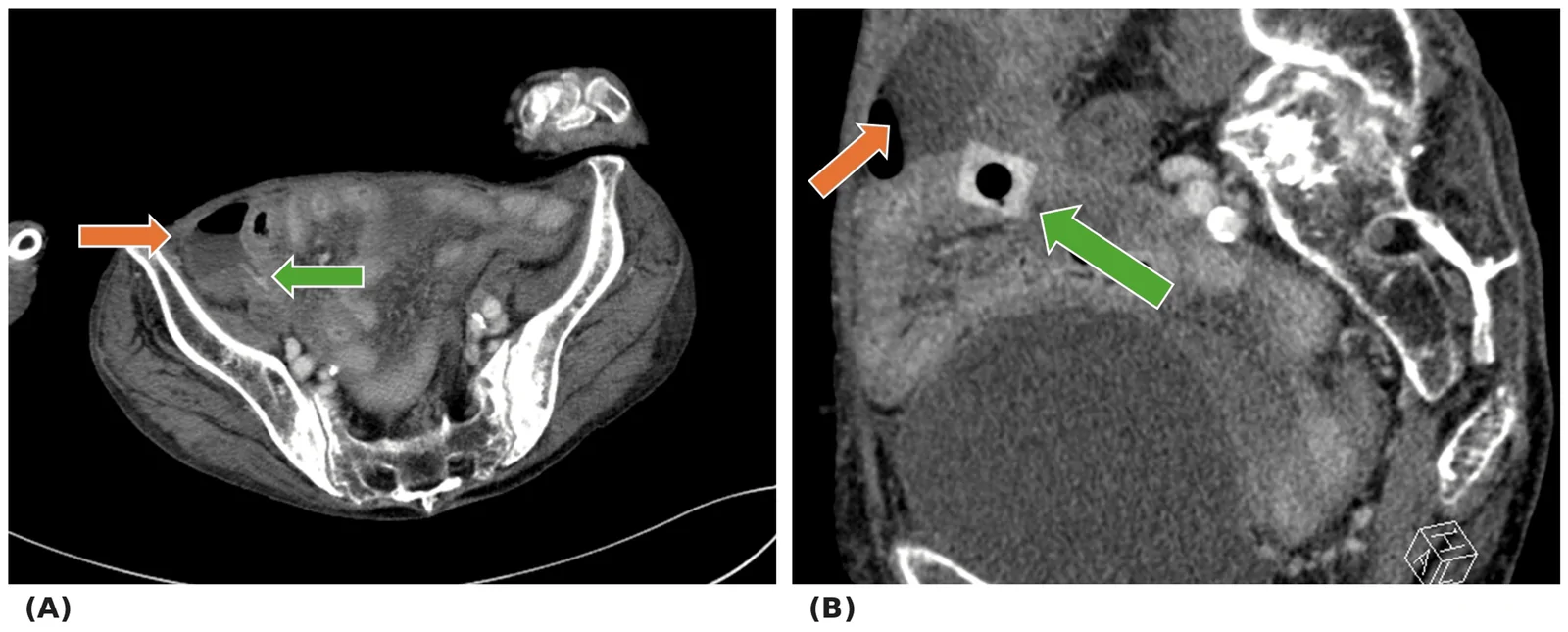
Contrast-enhanced abdominal computed tomography findings (A) Axial image. The orange arrow indicates the abscess cavity between the abdominal wall and the bowel in the right lower quadrant. The green arrow indicates the retained PTP within the ileum approximately 10 cm proximal to the ileocecal valve, identifiable as a high-density tablet surrounded by a low-density halo corresponding to the air pocket within the PTP dome. (B) Reconstructed image. The orange arrow again demonstrates the abscess cavity. The green arrow identifies the PTP, where its morphology is more clearly delineated on this projection. PTP: press-through package

Contrast-enhanced abdominal CT revealed a tablet retained within a PTP sheet, which appeared as a round, high-density object surrounded by a low-density halo, in the ileum approximately 10 cm proximal to the ileocecal valve [1,4], along with an abscess cavity between the abdominal wall and the bowel in the right lower quadrant and localized free air, indicating contained perforation (Figure 1).

**Figure 2:**
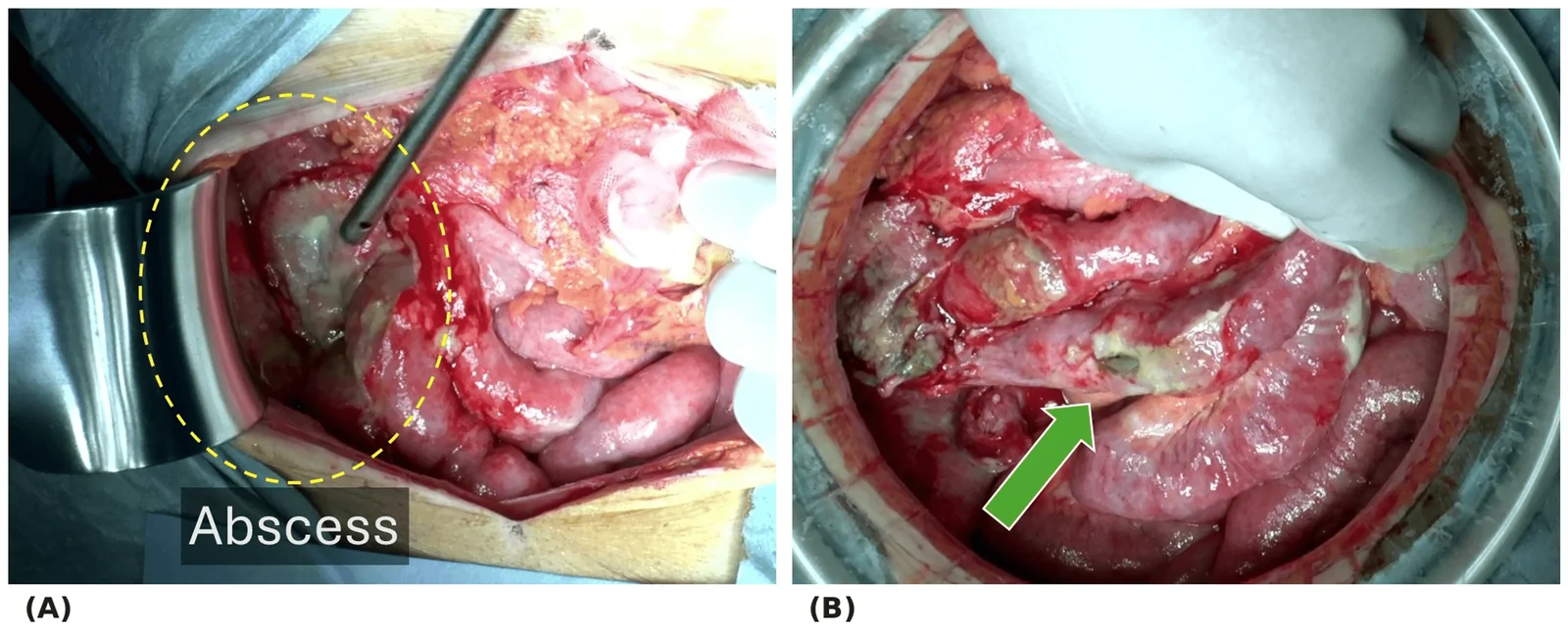
Intraoperative findings (A) The yellow dashed circle encircles the abscess cavity between the abdominal wall and the ileum at the perforation site. (B) The green arrow indicates the press-through package sheet, retained in its original form and partially extruding through the spindle-shaped ileal perforation.

Emergency laparotomy confirmed localized purulent peritonitis confined to the right lower quadrant. An abscess cavity between the abdominal wall and the bowel at the perforation site was identified (Figure 2A, yellow dashed circle). A spindle-shaped ileal perforation was observed, with an intact PTP sheet partially extruding through the defect (Figure 2B, green arrow).

**Figure 3:**
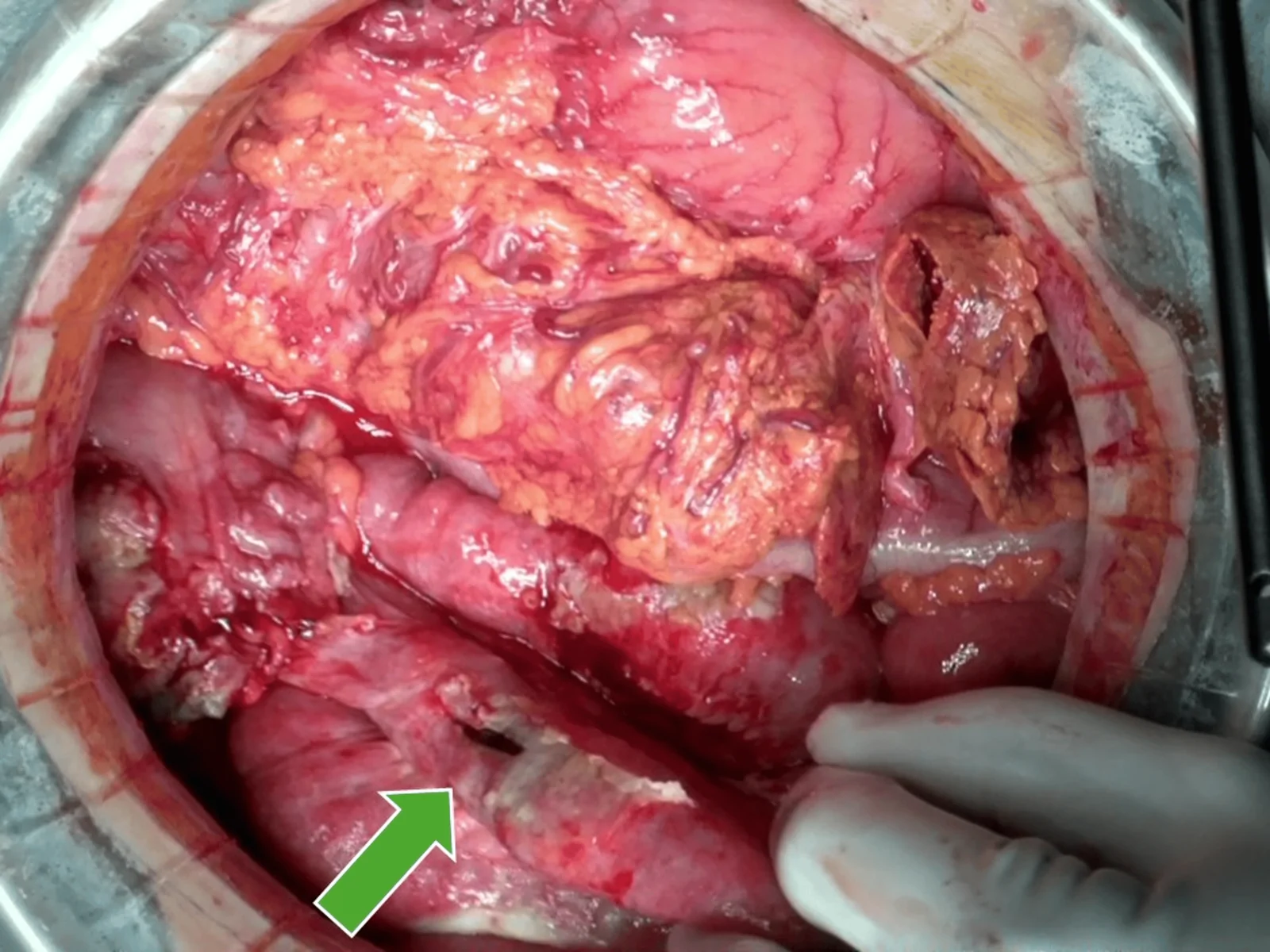
Intraoperative photograph of the perforation site The green arrow indicates the spindle-shaped perforation site in the ileum.

The perforation's anatomical location relative to the cecum, ascending colon, and terminal ileum is illustrated in Figure 3.

Primary suture repair with omental reinforcement and peritoneal lavage with drain placement were performed; bowel resection and stoma creation were unnecessary. The postoperative course was uneventful, and the patient was discharged on postoperative day 15 and was well during her final clinic visit.

## Discussion

The present case is notable for two reasons: First, a PTP was identified on CT despite the patient being unaware of having ingested it. Second, it highlights a high-risk demographic in whom occult PTP ingestion is easily overlooked: a very elderly woman with dementia who lived alone and managed her medication without supervision. In a previously reported case of PTP-induced ileal perforation published in this journal, a 75-year-old man underwent ileocecal resection [1]; in contrast, the small size of the perforation and the localized nature of the peritonitis in our patient permitted primary repair without bowel resection, avoiding the need for stoma creation in a very elderly patient.

CT is the investigation of choice when PTP ingestion is suspected. The characteristic CT finding of a round, high-density tablet enclosed within a low-density ring corresponding to the air-filled PTP dome has been documented in the literature [1,4]. A retrospective study of 61 patients reported CT detection rates of 97.8% for polyvinyl chloride/polyvinylidene chloride-based blister packs, supporting the utility of CT in this setting [6]. Plain radiography is unreliable for detecting retained PTPs, particularly after the tablet has dissolved [4]. Additionally, free intraperitoneal air, typically indicative of hollow viscus perforation, may be absent in cases of foreign body perforation in the small bowel [1,2]. In this case, CT not only confirmed the presence of the PTP but also identified the perforation site, abscess location, and extent of peritonitis, which facilitated a focused surgical strategy. When an elderly patient presents with acute abdominal pain of unclear etiology, prompt CT imaging should be performed, particularly if there is no history of foreign body ingestion, as this absence may result from cognitive impairment rather than indicating that ingestion did not occur.

Cognitive impairment substantially increases the risk of unwitnessed PTP ingestion. Patients with dementia may inadvertently swallow a PTP and be unable to reliably report it, making clinical suspicion the only prompt for further investigation [1,7-9]. This patient had several contributing risk factors: she lived alone without a caregiver to supervise medication administration; her only prescribed medication, diazepam, was dispensed in PTP format; and she had developed the habit of cutting individual tablets from the sheet with scissors, a practice intended to simplify dose management but results in single-tablet PTP units with sharp edges capable of causing intestinal perforation [1,2]. Current PTP design mandates perforation lines span at least two tablets to prevent manual separation into swallowable single units; however, scissors can easily bypass this safeguard. Strategies to mitigate this risk in cognitively impaired patients warrant proactive consideration. For patients receiving multiple medications, one-dose compounding, in which each scheduled dose is pre-packaged in a single sachet that eliminates PTP sheets, is an established preventive approach. For patients on a single agent, such as in this case, one-dose compounding is generally not indicated; in such situations, dispensing the medication in an alternative format, such as a tablet bottle or pharmacist-prepared strip packaging, may be the most practical method of removing the ingestion hazard.

This case report has some limitations. First, the dementia subtype was not formally characterized; the diagnosis was based on clinical assessment without standardized neuropsychological testing. Second, histopathological examination of the perforation site was not conducted, as primary repair was performed instead of resection. Third, structured long-term follow-up data are unavailable. Fourth, the inherent constraints of a single case report limit generalizability. Furthermore, biopsy of the perforation edges was not performed; although the intraoperative findings were consistent with foreign body perforation and no macroscopic features suspicious for malignancy were identified, histological examination of the perforation margins would have been preferable to exclude alternative pathology.

## Conclusions

The characteristic CT appearance of a retained PTP confirmed the diagnosis of ileal perforation in an elderly patient with dementia who had no recollection of the ingestion. In this case, CT not only established the diagnosis but also guided the operative approach by delineating the perforation site and the extent of peritonitis. Primary repair without bowel resection was achieved owing to the small size of the perforation (10 mm) and the localized nature of the peritoneal contamination. In cases of acute abdomen in elderly patients with cognitive impairment, PTP ingestion should be considered in the differential diagnosis, regardless of whether an ingestion history can be elicited.
